# The Relations of the Medical Profession to the Use and Abuse of Alcoholic Liquors

**Published:** 1886-07

**Authors:** Joseph P. Logan

**Affiliations:** Atlanta, Georgia


THE ATLANTA AND MEDICAL SURGICAL JOURNAL.

VOL. III. JULY, 1886. No. 5.

### Original Communications.

### THE RELATIONS OF THE MEDICAL PROFESSION TO THE USE AND ABUSE OF ALCOHOLIC LIQUORS.

READ BEFORE THE MEDICAL ASSOCIATION OF GEORGIA, AT AUGUSTA, 21ST OF APRIL, 1886, BY JOSEPH P. LOGAN, M. D., ATLANTA, GEORGIA.

Though it is anticipating to some extent what I desire to present to this body, I have regarded it as not inappropriate to say in the outset that while I have no adverse word for what are called temperance or total abstinence organizations, I do not speak as their representative, having no connection with them and not being bound by any pledge to advocate their views or practice their prohibition, but what I have to say upon this occasion is from a purely professional standpoint, and is intended to be a discussion of the use and abuse of alcoholic liquors as strictly related to the science and art of medicine. But having said so much, I cannot ignore the fact that the medical profession is regarded, to a very large extent, as furnishing one of the most serious obstacles in the way of the great moral reform, which is agitating the world in connection with the effort to suppress the liquor traffic, and it therefore seems to be appropriate to consider seriously the questions, to what extent, if at all, this is true, and if true, is there sufficient justification in any therapeutic advantage for the existing use of alcoholic liquors by medical men? From recent reflections as to the facts in the case, I am disposed to admit the correctness of the impeachment referred to, and to question whether there is any adequate compensation for the support which is now given to this legalized evil through the demand of the physician for alcoholic liquors for therapeutic purposes. Indeed, I am strongly inclined to the conclusion that no greater delusion has been imposed upon the medical profession or the world than the idea that there is anything really beneficial in alcoholic liquors, in either health or disease, that cannot be substituted. It can hardly now be doubted that alcohol is the real principle upon which medical reliance is placed in all these compounds which go under the general term of spirituous liquors, and as the result of the most careful and scientific observations upon the part of a number of competent observers as to the effects of alcohol upon the human body in health, it may be stated with absolute certainty that it is antagonistic to all physiological operations, and furnishes no aid whatever to any vital process; but, on the contrary, the normal activities are diminished, as strikingly manifested by a depressed temperature and a decreased muscular power.

Dr. N. S. Davis, the father of the organized medical profession in the United States, as the result of his own careful experiments and observations, combined with the experimental researches of a number of medical men in England, France and the United States, has formulated the following propositions:

1st. That alcohol, when taken diluted in the form of fermented or distilled spirits, is rapidly absorbed without change, carried into the blood, and with that fluid brought into contact with every structure and part of the human body.

2nd. That while circulating in the blood its presence retards those molecular or atomic changes by which nutrition, disintegration and secretion are maintained and the phenomena of life continued.

3rd. That its presence retards the elimination of waste matter, impairs nervous sensibility, lessens muscular excitability and lowers the temperature of the body.

4th. That a part at least (and from the testimony of other observers, I would say the whole) of the amount taken is finally eliminated or thrown out of the system with the excretions without having undergone any appreciable chemical change. As he further remarks, "these facts are as well established as any in the domain of physiology or in the whole field of natural science, and they point, with all the clearness and force of a mathematical demonstration, to the conclusion that alcohol is in no sense food, neither furnishing material for the tissues nor fuel for combustion, nor yet generating either nervous or muscular power." But, on the other hand, I would remark that it is essentially a poison, antagonizing some of the most important interstitial changes upon which the vital forces are dependent, and is the fruitful source, when habitually used, short of intoxication, of the most serious and fatal diseases which the physician is constantly called upon to treat, which can be exhibited most fully by numerous facts readily drawn from the standard literature of the profession. In reply to the second question, as to any compensating advantage to the human family in the relief of disease, for the incalculable injury inflicted in the production of disease and the demoralization of humanity by the countenance given to its use and traffic through its recommendation as a medicinal agent, I would say that there is, in my judgment, no conclusive evidence that alcohol has any more power in rectifying morbid processes and removing disease than it has in promoting the vital processes in a state of health. I will go still farther and assert that alcoholic liquors, whatever may be the undemonstrated advantages, have no absolutely defined place in therapeutics and cannot be proven to occupy any distinctive or legitimate position as agents for the treatment of disease, that there are the most discordant, loose and undefined views in our approved medical works as to their application, and practically no exact therapeutic rules clearly established as to their *modus operandi* in special diseases, or as to the quantity in which they are to be administered, and thus lacking all recognized essential elements of approved and reliable medicinal agents. While this is to a great extent true in regard to simple alcohol, it is to a much greater extent true in reference to brandy, whisky, rum, gin, wine and the legion of malt liquors, all of which are prescribed by medical men, and the people, following their example, with the most reckless disregard to the systematic and definite purpose supposed to properly belong to the use of agents which have obtained a fixed place in the materia medica. To which may be added the notorious adulteration of all forms of the liquors referred to, destroying reliance upon them as medicinal agents, even to the limited extent of which there may be any proof they deserve even when pure, from the admixture of injurious and in many instances of poisonous ingredients, as well as from the varying and uncertain quantities of alcohol which they contain.

While I have been making progress in the direction of the conviction that alcohol and its admixtures, used as a beverage, may possibly be dispensed with as medicinal agents, I will only go so far at present as to say that their use should be discontinued, unless some more definite knowledge of their proper application can be presented. Some of our most enlightened medical men are now having their attention especially directed to a consideration of the question how far, if at all, alcohol is beneficial as a remedy in certain diseases for which it has long been prescribed.

I have for a number of years condemned its use in consumption, as not only not beneficial, but as absolutely injurious, by the aggravation of the malnutrition, which is one of the most conspicuous and essential elements of the disease; and it is now asserted with confidence, to which I am strongly disposed to accede, that its use is really in some cases the cause of certain forms of the disease. The tendency manifestly is, among some of our most intelligent medical observers, to abandon its use in this disease, and thus drive it out from one of its principal strongholds. I think I can say with perfect candor, in a very laborious and extended professional life, I cannot recall a half dozen instances in which I really believed that life had been saved by alcoholic liquors, and the inclination of my mind is strongly toward the conviction that the time will come, at no very distant day, when alcohol in any of its forms will no longer be recognized as a medicinal agent, beyond its value as a menstruum, extractive and preservative agent, or as the source from which may be obtained some one or more innocuous and possibly beneficial elements. And even before the good time when the physician will no longer prescribe it, the government will no longer tax and thus protect and foster the enormous evil involved in its traffic. We are now appealed to and relied upon to stand by the druggists in enabling them to retain their privilege of vending liquors under cover of supplying the needs of medical men, by which they have brought so much discredit upon themselves and upon the medical profession, until we have become largely identified with them in the deadly traffic, in which, with honorable exceptions, they are so largely engaged. To the pharmaceutist and prescriptionist who legitimately pursues his vocation as an aid to the physician, of which I regret to say there are but few, we should feel indebted, and they should have all the facilities which are necessary to enable them to comply with the proper demands upon them; but the originator and vendor of the many nostrums largely containing liquor, and which to a great extent furnishes the facility for their sale, and who is really one of the most formidable enemies of the physician, and whose business is largely dependent upon the extent to which he can unsettle the confidence of the public in him, surely has no claim upon us.

While individually I do not hesitate to say that I would greatly rejoice in the exchange of the present status of alcoholic preparations for their entire extirpation, believing, as I do most solemnly, that their use, even by physicians in the unsettled and unscientific manner in which they are largely prescribed, is an infinite evil, and we are not in any way compensated by whatever of good is derived therefrom; yet I fully recognize the fact that many intelligent and reliable physicians, who have my respect, regard alcoholic liquors in some form as important to them in the discharge of their professional duties. I fully recognize the fact, whatever I may individually think of the present use of alcohol or anticipate of the future, as already indicated, that the time has not arrived, and may never come, when the State can deny to the physician the use of simple alcohol, based as it is upon his perfect right to the exercise of his best judgment with the most perfect independence, within the limits of professional approval, in the selection of remedies for the treatment of disease. But if it is indispensable and must be furnished, let it be done under such restrictions and under such supervision as obtain in regard to the sale of other poisons, and as will lessen to the greatest possible degree the liability to a perversion of the privilege, even if it should be necessary to appoint and hold accountable its own agents. In the language of the distinguished Dr. Richardson, "On the other side, in common fairness to scientific progress, the professors of healing ought so to prescribe alcohol that nothing shall be wanting in accuracy of prescription; the exact quantity, the exact quality, the exact purity of the alcohol ought to be known and due provision made to insure what is right in respect to quantity, quality and purity. To prescribe wine without knowing whether the specimen of wine contains ten, fifteen or twenty per cent. of alcohol; to prescribe spirits without knowing the percentage of alcohol, or whether all the alcohol in the spirits is ethylic alcohol or a mixture of alcohols; to prescribe either wines, spirits or ales without asking whether other chemical bodies than alcohol are or are not present in them, is not prescribing at all. It is the duty of the physician to order in fact the absolute remedy, to define the dose and to direct the administration with the same care as if he were prescribing opium, arsenic, chloral or other poisonous or active remedy." While no man can gainsay the absolute fairness of such requirements, or deny the imperative obligation involved, it is an undeniable and lamentable fact that no such practice obtains and no such knowledge exists as would enable the physician to come up to the full measure of duty as here set forth. What is the inevitable obligation arising from such a state of facts? Manifestly either to establish the use of alcohol as a medicinal agent upon a scientific basis, with its special and accurate therapeutic details and applications, or abandon its use. But notwithstanding a large portion of the profession are still bound by the foggy traditions of the past, the light is breaking and the evidences of progress are not lacking, and ere many years the reproach upon the profession connected with alcohol will have disappeared.

About two hundred physicians and surgeons of New York, Brooklyn and vicinity recently signed the following medical declaration: "We are of the opinion that the use of alcoholic liquor as a beverage is productive of a large amount of physical disease, that it entails diseased appetites upon offspring, and that it is the cause of a large percentage of the crime and pauperism of our cities and country. We would welcome any judicious and effective legislation—state and national—which should seek to confine the traffic in alcohol to the legitimate purposes of medical and other sciences, art and mechanism."

Over two thousand physicians of Great Britain declared, "That total and universal abstinence from alcoholic beverages of all sorts would greatly contribute to the health, the prosperity and happiness of the human race." Dr. B. W. Richardson, of London, says: "I hold that those who abstain from alcohol have the best digestion, and that more instances of indigestion, of flatulency, of acidity and of depression of mind and body are produced by alcohol than by any other single cause. It is an agent as potent for evil as it is helpless for good. It begins by destroying, it ends by destruction, and it implants organic changes which progress independently of its presence, even in those who are not born." Dr. James Edmunds, of England, says: "It is admitted by every one that alcohol is the cause of more than half of the insanity we have. It is a fact which cannot be disputed that diseases of the liver, diseases of the lungs, diseases of the tissues of the body, are induced directly by the use of alcohol." Individually, I have been long convinced that a large proportion of mortality in ordinarily curable disease was due to an exhaustion of reserved vitality as the result of the habitual and perhaps not intoxicating use of alcohol. Dr. Willard Parker, of New York, says upon this point: "Alcohol has no place in the healthy system, but is an irritant poison, producing a diseased condition of body and mind. It has been demonstrated that the use of alcohol, when employed moderately, as many young men often use it, as they think, with impunity, makes the average of life thirty-five and a half; while that of non-users reached an average of sixty-four and one-sixth years, or a difference of about twenty-nine years to each individual." Sir Henry Thompson says: "I have long had the conviction that there is no greater cause of evil, moral and physical, than the use of alcoholic beverages. I do not mean by this that extreme indulgence which produces drunkenness. The habitual use of fermented liquors to an extent far short of what is necessary to produce that condition—and such as is quite common in all ranks of society—injures the body and diminishes the mental power to an extent which I think few people are aware of. Such, at all events, is the result of observation during more than twenty years of professional life, devoted to hospital practice and to private practice in every rank above it. Thus I have no hesitation in attributing a very large proportion of some of the most painful and dangerous maladies which come under my notice, as well as those which every medical man has to treat, to the ordinary and daily use of fermented drink, taken in the quantity which is conventionally deemed moderate."

James R. Nichols, M. D., the distinguished chemist of New England, says : "That if the natural vinous fermentative process should cease and the art of distillation become a 'lost art,' not a life would be sacrificed in consequence, not a case of disease would be retarded in the cure, not a pain would be aggravated and not one of the art processes suffer detriment. Vast changes have occurred in medicine, pharmacy, chemistry, and in all art processes, and it is well to look about and ascertain our true position as regards the present necessity for the use of alcohol. Since the active alkaloidal and resinoidal principles of roots, barks and gums have been isolated and put in better and more convenient forms, there is no longer need of alcoholic tinctures and elixirs. Laudanum, which is a tincture of opium, might be banished from the shelves of every apothecary, as it is not needed It is now known that the valuable narcotic and hypnotic principles of opium are contained in certain crystalline bodies which can be isolated and used in minute and convenient forms and that they can be held in aqueous solutions. Alcohol is no longer needed to hold the active principles of opium, Peruvian bark or other indispensable drugs. The catalogue of modern remedies is almost endless, and many of them hold alcohol in some form, but every intelligent physician knows that ninety per cent. of these alleged remedies have little or no intrinsic value. The nostrums of the quack, the bitters, elixirs, cordials, extracts, etc., nearly all contain alcohol, and this is the ingredient which aids in their sale. The whole unclean list might with advantage be thrown to the fishes."

Dr. Charles Jewett, of Connecticut, says: "Unless the medical men of our country speedily and radically change their present practice in relation to the prescription of intoxicating liquors for well-nigh half of the diseases they are called to treat, one of two important results is sure to follow, either the educated portion of our people, becoming more and more enlightened in relation to the physiological action of alcohol, will reject the teachings and prescriptions of the doctors, and lose confidence in them, or if the present order of things shall continue, all our efforts to lessen materially the burdens and evils of intemperance will prove vain, and the great curse will be perpetuated through generations yet to come." Thus we see the great upheaval which is beginning in the medical profession, the result of which, in my judgment, will produce an entire revolution in the present practice of physicians in the use of alcohol. What part shall we bear in this great professional reform upon which so much that is vital to the race depends? Shall we throw obstacles in the way? Shall we stand idly by, or pursuing the same old beaten track refuse to recognize the facts which are becoming so plain that "he who runneth may read," or shall we come up to the full measure of our opportunities and obligations as physicians, as conservators of the public health, as philanthropists, as citizens, as men who acknowledge our accountability to conscience and to God? Our own State is in the midst of the throes of a great effort to break the chains of a worse than Egyptian bondage. We cannot wrap ourselves in professional dignity if we would. We have no right to be neutral. We will not and should not be allowed to be. We wield an influence which will be potent for good or evil. The advocates of the liquor traffic are constantly endeavoring to marshal us among their forces upon the plea that the Legislature is proposing to dictate to us what medical agents we shall use, and to interfere with the right of the people to have alcoholic liquors for medicine is a usurpation of power. Whether the law which the advocates of prohibition are seeking to have adopted by the people is perfect or free from objections or not, we must either accept it or allow the traffic in liquor to continue. While I do not advocate any organized action upon the part of the medical profession in favor of the present legislation, I shall at least hope that as individuals we shall not withhold whatever aid may be in our power to those who would forge chains for, and make disreputable the traffic in that which is now made respectable and protected by law, which causes a large proportion of the disease for which it is prescribed, which as medical men we are compelled to admit creates and entails idiocy and insanity, and crowds our hospitals and insane asylums with its wretched victims.

